# Why is Mini-Mental state examination performance correlated with estimated premorbid cognitive ability?

**DOI:** 10.1017/S0033291716001045

**Published:** 2016-07-05

**Authors:** D. Dykiert, G. Der, J. M. Starr, I. J. Deary

**Affiliations:** 1 Centre for Cognitive Ageing and Cognitive Epidemiology, University of Edinburgh, EdinburghUK; 2 Department of Psychology, University of Edinburgh, EdinburghUK; 3 Medical Research Council/Chief Scientist Office Social and Public Health Sciences Unit, University of Glasgow, Glasgow, UK; 4 Geriatric Medicine Unit, University of Edinburgh, EdinburghUK; 5 Alzheimer Scotland Dementia Research Centre, University of Edinburgh, EdinburghUK

**Keywords:** Mini-Mental State Examination, National Adult Reading Test, premorbid cognitive ability, Wechsler Test of Adult Reading

## Abstract

**Background:**

Tests requiring the pronunciation of irregular words are used to estimate premorbid cognitive ability in patients with clinical diagnoses, and prior cognitive ability in normal ageing. However, scores on these word-reading tests correlate with scores on the Mini-Mental State Examination (MMSE), a widely used screening test for possible cognitive pathology. This study aimed to test whether the word-reading tests’ correlations with MMSE scores in healthy older people are explained by childhood IQ or education.

**Method:**

Wechsler Test of Adult Reading (WTAR), National Adult Reading Test (NART), MMSE scores and information about education were obtained from 1024 70-year-olds, for whom childhood intelligence test scores were available.

**Results:**

WTAR and NART were positively correlated with the MMSE (*r* ≈ 0.40, *p* < 0.001). The shared variance of WTAR and NART with MMSE was significantly attenuated by ~70% after controlling for childhood intelligence test scores. Education explained little additional variance in the association between the reading tests and the MMSE.

**Conclusions:**

MMSE, which is often used to index cognitive impairment, is associated with prior cognitive ability. MMSE score is related to scores on WTAR and NART largely due to their shared association with prior ability. Obtained MMSE scores should be interpreted in the context of prior ability (or WTAR/NART score as its proxy).

## Introduction

Estimating a person's peak prior level of cognitive functioning is useful in clinical and research settings. Estimated peak prior cognitive level can provide a baseline from which to assess the severity of cognitive impairment following brain trauma, psychiatric or neurological disorder, or the degree of cognitive decline associated with non-pathological ageing. Estimating prior ability using cognitive tests is challenging because a score on a test is a function of the peak prior trait level of cognitive functioning and of any decline from that level. Therefore the same low score might be obtained by a person with low prior ability level and no decline and by a person with a higher level of initial ability who suffered a degree of cognitive decline. Prior ability is often estimated using tests which tap crystallized cognitive abilities (Horn & Cattell, 1967), which remain relatively unaffected even in the presence of a degree of cognitive impairment (Nelson & McKenna, 1975). In an affected individual, the estimated prior ability can be compared with performance on fluid-type tests that are sensitive to cognitive impairment. There is evidence that this estimated prior *v.* current fluid cognitive ability provides an approximate but valid estimate of actual cognitive change in healthy older people (Deary *et al.* 2004). However, the validity of this method would be compromised if the premorbid ability tests were sensitive to the degree of cognitive impairment, which has been mooted in the literature (e.g. O'Carroll *et al.* 1995; McFarlane *et al.* 2006). If this is the case, then premorbid ability could be underestimated and, consequently, so would the amount of cognitive impairment that had occurred. In the present study we tested the degree to which two tests used to estimate premorbid cognitive ability are associated with a measure of cognitive impairment.

The Wechsler Test of Adult Reading (WTAR) and the National Adult Reading Test (NART) are often used to estimate premorbid (before a clinical diagnosis or brain injury) or prior (before the onset of normal ageing) cognitive ability. Both of these tests utilize the irregular grapheme-phoneme mappings or stress patterns in English words, together with the relative stability of reading ability in the face of cognitive deterioration (e.g. Nelson & McKenna, 1975). The reasoning behind these tests assumes that (*a*) irregular words will tend to be pronounced incorrectly if encountered for the first time in a written form; (*b*) the knowledge of correct pronunciation will remain even in the presence of a degree of cognitive decline; and (*c*) people with higher cognitive ability will have learned a larger number of less common words. Both the WTAR and NART require participants to read a list of irregular words, so in this paper they will sometimes be collectively referred to as the reading tests.

## Reading tests and cognitive impairment

Scores on both WTAR and NART have been shown to be valid estimators of prior (Crawford *et al.* 2001; Deary *et al.* 2004; McGurn *et al.* 2004; Dykiert & Deary, 2013) and premorbid (McGurn *et al.* 2004; Green *et al.* 2008) cognitive ability. However, there have been reports of positive moderate correlations between scores on the reading tests and on the Mini-Mental State Examination (MMSE, Folstein *et al.* 1975), a widely used screening test for potential cognitive impairment [*r* = 0.56 in AD patients (Patterson *et al.* 1994); *r* = 0.51 in a group with dementia of mixed aetiology (McGurn, *et al.* 2004); and *r* = 0.49 in healthy older adults (Starr *et al.* 1992)].

The positive correlation between the reading tests and MMSE scores might mean that performance on the reading tests, which is meant to be stable in cognitive decline, is actually affected in cognitive impairment. Some existing research seems to support this possibility. For example, differences in both WTAR and NART scores were reported for patients with varying dementia severity, who were generally well-matched and expected to have similar premorbid ability levels (Stebbins *et al.* 1990; O'Carroll *et al.* 1995; McFarlane *et al.* 2006). Further, NART performance appears to improve during recovery from traumatic brain injury (Riley & Simmonds, 2003). However, the same was not found for the WTAR (Green *et al.* 2008).

One study of healthy older people which had rarely available data on actual premorbid or prior ability (IQ measured in childhood) provided an alternative explanation for the NART–MMSE association (Crawford *et al.* 2001). It suggested that the apparent sensitivity of the reading tests to the severity of cognitive impairment (the NART–MMSE correlation) may be caused by the MMSE's being associated with prior cognitive ability level. In that study a significant correlation in older people between NART and MMSE (which might have indicated NART's sensitivity to the degree of cognitive impairment) was abolished after controlling for actual prior (childhood) mental ability. It suggested that the relationship between NART and MMSE largely reflects the variance shared between these measures and prior cognitive ability, rather than suggesting that NART scores depend on the degree of cognitive impairment. To our knowledge, no study has investigated WTAR in this respect.

## Aims of the study

The present study aimed to investigate whether the WTAR is related to MMSE score, independently of actual prior cognitive ability. We did this by testing whether the correlation between WTAR and MMSE in a large sample of healthy older people is substantially attenuated when childhood IQ is controlled. We also aimed to replicate the finding of Crawford *et al.* (2001) for the NART–MMSE association in our sample. Moreover, we tested whether education accounts for some of the association between WTAR–NART and MMSE.

## Method

### Participants

Participants were members of the Lothian Birth Cohort 1936 (LBC1936), an ongoing longitudinal study of healthy ageing. The study has been described in detail previously (Deary *et al.* 2007, 2012). In brief, the cohort was drawn from participants in the Scottish Mental Survey of 1947 (SCRE, 1949), which administered the same intelligence test to almost all children born in 1936 and attending Scottish schools on 4 June 1947. The LBC1936 cohort had adult tests administered for the first time at a mean age of ~70 years. Their intelligence test scores at age 11 years were made available. To date, three waves of testing have been completed, at mean ages of 70, 73, and 76 years. Each wave comprises an interview during which demographic and health data are collected, and a cognitive battery and physical tests are administered by psychologists and research nurses, respectively. The main analyses in the present study use age 11 intelligence test scores and data from wave 1 (mean age = 69.53, s.d. = 0.84 years). Subsequent testing waves were used for a sensitivity analysis.

At wave 1 there were 1091 participants (548 men/543 women) who took part in the study. Age 11 IQ data were not available for 63 of them and a further four had missing data on the variables of interest (see below) at wave 1. Excluding them left the working sample of 1024.

### Cognitive tests, education and dementia diagnosis

#### Age 11 IQ

A modified version of Moray House Test (MHT) no. 12 (SCRE, 1933) was used to measure IQ at age 11. The MHT scores have been validated against those on the Stanford–Binet and on the Terman–Merrill intelligence tests, with high correlations *r* ≈ 0.8 (SCRE, 1933, 1949). The MHT is a timed test (45 min) comprising 71 items of different types, including, following instructions, same-opposites, word classification, analogies, arithmetic, spatial, or cypher decoding. The maximum score is 76. It was group-administered at schools on one day (4 June) in 1947. To eliminate variance in MHT scores due to age differences at the time of its administration, the raw MHT scores were regressed on age (in days) on 4 June 1947. The residuals were then scaled to the mean of 100 and s.d. of 15 to create IQ-type scores.

#### Reading tests

Two reading tests were administered that are used to estimate premorbid/prior cognitive ability: the WTAR (Psychological Corporation, 2001) and the NART (Nelson & Willison, 1991). Both tests use a list of 50 irregular words, arranged in order of difficulty, from simple (*again, ache*) to difficult (*insouciant, syncope*). Participants are required to read these words aloud and a trained tester assesses the correctness of the pronunciation. We used the number of correct responses as a score on the WTAR and NART, respectively.

#### The MMSE

The MMSE (Folstein *et al.* 1975) was administered to participants at each wave. It is often used as a screening test for signs of cognitive impairment. It tests various abilities, including attention, memory, language and comprehension, figure drawing, and basic orientation. Maximum score is 30 and a score below a cut-off of 24 is often used to indicate possible dementia (Tombaugh & McIntyre, 1992).

#### Education

The principal educational attainment variable was a self-reported highest educational qualification obtained, which was coded as follows: 0, no qualification; 1, O-level or equivalent; 2, A-level or equivalent; 3, semi-professional/professional qualification; and 4, degree. Self-reported number of years in full-time education was also recorded. Main analyses were performed with highest qualification as an indicator of educational attainment. They were later repeated with years of education entered instead of highest qualification as an alternative indicator.

#### Dementia diagnosis

Self-reported history of dementia was obtained during an interview at every wave of testing.

### Statistical analysis

Basic analyses were performed in IBM SPSS Statistics, v. 19 (IBM Corp., USA). Auxiliary analyses were performed in R software v. 3.2.1 (https://cran.r-project.org/bin/windows/base/old/3.2.1/). Differences between correlations (Steiger, 1980) were tested using r.test command in package ‘psych’ (Revele, 2015). Sensitivity analyses using Poisson regressions were implemented using glm command in ‘stats’ package (R Core Team, 2015).

## Ethical standards

The authors assert that all procedures contributing to this work comply with the ethical standards of the relevant national and institutional committees on human experimentation and with the Helsinki Declaration of 1975, as revised in 2008.

## Results

Descriptive statistics and correlations between WTAR, NART, MMSE, and age 11 IQ are shown in Table 1. The high positive correlation between WTAR and NART and between the reading tests and age 11 IQ were reported previously on a subsample of the LBC1936 who attended two waves of testing (Dykiert & Deary, 2013) and they are near-identical to the ones found here.

**Table 1.** Descriptive statistics and inter-correlations of cognitive tests scores and education levels (N = 1024)

	Descriptive statistics				Correlations				
	Mean	s.d.	Min	Max	NART	MMSE	Age 11 IQ	Education (years)	Qualification
WTAR	41.13	7.04	14	50	**0.89**	**0.42** (**0.23**)	**0**.**67**	**0.49** (**0.31**)	**0.55** (**0.34**)
NART	34.55	8.06	10	50		**0.41** (**0.20**)	**0.69**	**0.52** (**0.35**)	**0.57** (**0.37**)
MMSE	28.83	1.37	22	30			**0.40**	**0.19** (0.03)	**0.23** (0.05)
Age 11 IQ	100.02	15.01	38.48	129.88				**0.42**	**0.49**
Education (years)	10.72	1.12	7	14					
Qualification	1.66	1.30	0	4					
Age at testing in childhood (years)	10.94	0.28	10.42	11.42					
Age at testing in older age (years)	69.53	0.84	67.61	71.30					

WTAR, Wechsler Test of Adult Reading; NART, National Adult Reading Test; MMSE, Mini-Mental State Examination.

Values in parentheses show partial correlation coefficients after controlling age 11 IQ.

Education represents number of years in full-time education; Qualification is the highest qualification obtained.

All correlations in bold are significant at *p* < 0.001.

MMSE correlated moderately with both WTAR (*r* = 0.42, *p* < 0.001) and NART (*r* = 0.41, *p* < 0.001). We remind the reader that these were all tested in older age. MMSE in older age was positively correlated with MHT score at age 11 (*r* = 0.40, *p* < 0.001), and with the highest qualification obtained (*r* = 0.23, *p* < 0.001) and the number of years in full-time education (*r* = 0.19, *p* < 0.001). WTAR and NART were both positively correlated with educational variables, with correlation coefficients between 0.49 and 0.57 (all *p* < 0.001).

## Does prior IQ account for MMSE–WTAR/NART correlations?

To test whether the correlations between the reading tests and MMSE reflect their shared sensitivity to differences in prior cognitive ability, we computed partial correlations between WTAR and MMSE and between NART and MMSE, controlling for age 11 IQ. Partialling out actual prior ability (age 11 IQ from the MHT test) reduced the correlations of WTAR and NART with MMSE. The partial correlation between WTAR and MMSE was *pr* = 0.23, *p* < 0.001, reduced from 0.42. This is a 70% reduction in the shared variance (*R*^2^) between WTAR and MMSE, from 17.6% to 5.3%. The partial correlation between NART and MMSE was *pr* = 0.20, *p* < 0.001, reduced from 0.41. This is a 76% reduction in the shared variance (*R*^2^) between NART and MMSE, from 16.8% to 4.0%. A formal test for comparing correlations (Steiger, 1980) confirmed that the WTAR–MMSE and NART–MMSE correlations were significantly as well as substantially attenuated by partialling out prior ability (WTAR: *z* = 9.40, *p* < 0.001; NART: *z* = 9.98, *p* < 0.001).

## Does education account for MMSE–WTAR/NART correlations?

All variables of interest (WTAR, NART, MMSE, age 11 IQ) were significantly related to education (Table 1). We investigated the role of education in associations between scores on the reading tests and on the MMSE. An adjustment for highest qualification obtained resulted in an attenuation of WTAR–MMSE and NART–MMSE correlations, but to a lesser degree than was seen with the before-mentioned childhood IQ adjustment. Partial correlation coefficients were *pr* = 0.36 and *pr* = 0.34, respectively, down from 0.42 and 0.41. These are 27% and 31% reductions, respectively, in the shared variance (*R*^2^) between WTAR–NART and MMSE (from 18/17% to 13/12%). Both were significant at *p* < 0.001 and significantly different from their respective zero-order correlation coefficients (WTAR: *z* = 3.97, *p* < 0.001; NART: *z* = 4.33, *p* < 0.001).

## To what extend do education *and* childhood IQ combined account for MMSE–WTAR/NART correlations?

Partial correlation coefficients, after controlling for both highest qualification and prior IQ simultaneously (WTAR: *pr* = 0.23; NART: *pr* = 0.19), were almost identical to those obtained after controlling for prior IQ alone (WTAR: *pr* = 0.23; NART: *pr* = 0.20). This suggests that education explained almost no variance in the WTAR–MMSE and NART–MMSE associations beyond that explained by age 11 IQ.

## Predicting MMSE from WTAR and NART scores

Because MMSE scores are related to childhood IQ, and because most of the MMSE's variance that is shared with childhood IQ is also shared with scores on reading tests, it might be possible to predict the ‘expected’ MMSE scores for a given level of prior or premorbid cognitive ability from scores obtained on WTAR or NART (see Crawford *et al.* 1992, for a similar approach in predicting verbal fluency performance). The expected MMSE scores, predicted from regression equations built on our sample, are presented in Table 2. We compared these predicted MMSE scores with the actual MMSE scores obtained. Mean discrepancy from the predicted MMSE was 0.08 (s.d. = 1.30) for the WTAR-predicted MMSE and 0.10 (s.d. = 1.27) for the NART-predicted MMSE. For both discrepancy scores calculated using WTAR and NART, a score of -4 or lower (i.e. an obtained MMSE score that is ⩾4 points below the predicted MMSE score) was obtained rarely, by only 1.5% of our sample. We suggest that this level of discrepancy might be used in screening participants or clients for possible cognitive decline or impairment.

**Table 2.** Table for converting obtained WTAR and NART scores into predicted MMSE scores

Obtained WTAR score	Obtained NART score	Predicted MMSE score	Suggested MMSE cut-off
14–16		25	⩽21
17–20	10–12	26	⩽22
21–26	13–18	27	⩽23
27–35	19–28	28	⩽24
36–50	29–48	29	⩽25
	49–50	30	⩽26

WTAR, Wechsler Test of Adult Reading; NART, National Adult Reading Test; MMSE, Mini-Mental State Examination.

The ranges of WTAR and NART scores presented here each begin with the lowest score observed in our sample. No extrapolations are made beyond those lowest scores.

In practice, an individualized MMSE cut-off may be obtained by predicting MMSE score from WTAR or NART, using the conversion table (Table 2), and deducting 4 points. For example, for a person with a NART score of 17 (i.e. for a person with an estimated IQ of approximately 100; Nelson & Willison, 1991) the predicted MMSE would be 27 and the individualized cut-off, 27 – 4 = 23. Thus, for a person with an average IQ, the individualized MMSE cut-off would be ⩽23 (i.e. <24) which corresponds to the one widely used in current practice. However, for a person with a NART score of 10 (corresponding to an IQ of 95), the MMSE cut-off would be lower, ⩽22, and for people with very high IQs (scoring 49 or 50 on the NART), it would be ⩽26.

## Sensitivity analysis

Partial correlations are easily understood and have been used to test similar research questions in the past (e.g. Crawford *et al.* 2001). However, partial correlations assume that associations between variables are linear and that there are no interactions. This might not be the case, especially since we are dealing with MMSE, whose distribution deviates from normality. Because errors on the MMSE (i.e. reverse of an MMSE score) approximate a Poisson distribution, we performed sensitivity analysis, in which MMSE errors were modelled as a Poisson variate in a generalized linear model. We then calculated the reduction in deviance due to WTAR/NART before and after controlling for age 11 IQ. The results were very similar to those obtained using partial correlations: the reductions in deviance due to WTAR/NART after controlling for age 11 IQ were 75% and 78%, respectively. The reductions in deviance due to WTAR/NART after controlling for highest qualification obtained were 36% and 37%, respectively (highest qualification was not significantly associated with MMSE errors when a reading test score was also included in the model). When both qualification and age 11 IQ were controlled, the reductions in deviance due to WTAR/NART were 79% and 81%, respectively. These were only slightly greater than the reductions after controlling for age 11 IQ alone and highest qualification was not a significant predictor of MMSE in these models. We also tested interactions between predictors in each model of MMSE. None was statistically significant.

We repeated all analyses reported above replacing highest qualification with years in full-time education – an alternative, commonly used, measure of educational attainment. The pattern of results was the same.

**Fig. 1.**
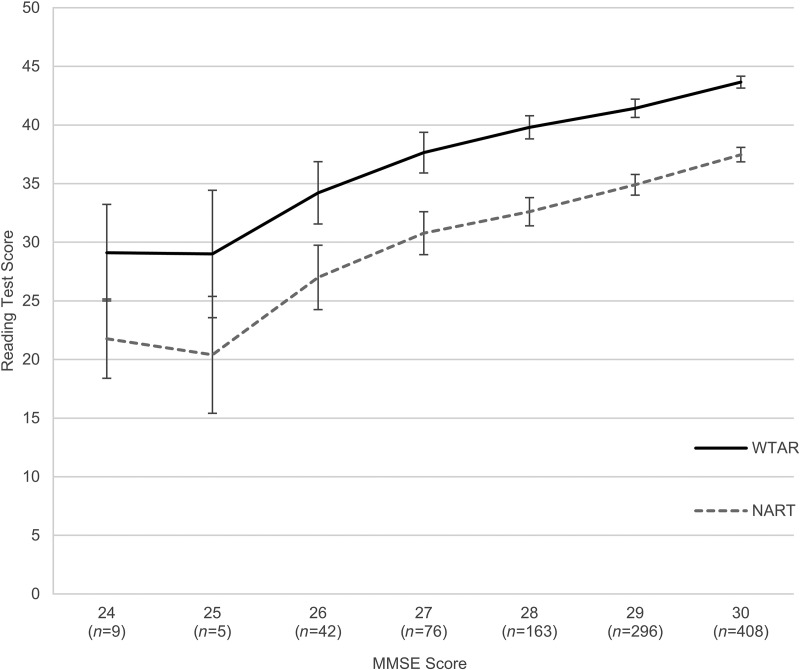
Average Wechsler Test of Adult Reading (WTAR) and National Adult Reading Test (NART) scores by Mini-Mental State Examination (MMSE) score. Error bars represent 95% confidence intervals.

We preformed another sensitivity analysis to investigate whether the relationships between MMSE, WTAR/NART and age 11 IQ might be driven by participants with greater degree of cognitive impairment. We excluded all cases with MMSE <24 or a diagnosis of dementia. To do this, we utilized the longitudinal data collected on the sample 3 and 6 years after the initial testing, at mean ages of 72.49 (s.d. = 0.72) and 76.24 (s.d. = 0.68). This way we could identify individuals whose performance at wave 1 might have been negatively affected by a preclinical cognitive impairment or dementia. In total, 25 participants were excluded with low MMSE or dementia diagnosis at baseline or during the 6-year follow-up (seven participants met the criteria already at wave 1, and further 18 at waves 2 and/or 3). The correlations between MMSE and WTAR/NART/age 11 IQ changed very little, indicating that they were not driven by individuals with lowest MMSE scores, and possible undiagnosed pathology, but operate even in what we defined as the normal range. The association is presented graphically in Fig. 1, in which mean scores on WTAR and NART are plotted against MMSE scores. There is a clear pattern of increasing WTAR and NART scores as a function of MMSE, which continues throughout the MMSE range.

## Discussion

WTAR and NART (which are used to estimate prior cognitive ability and would ideally not be affected by mild cognitive decline) were both positively correlated with MMSE (which is used to estimate cognitive decline and would ideally not be correlated with prior cognitive ability), with medium effect size, even in a relatively healthy sample. We tested whether controlling for cognitive ability at age 11 significantly attenuated the associations between the reading tests and MMSE. It did, with ~70% reduction in the shared variance between reading test and MMSE association. Considering that the correlations between childhood IQ and WTAR/NART are not perfect estimates – there is error of measurement in the variables – the extent of this already large attenuation is probably an underestimate. Controlling for education also attenuated the associations between the reading tests and the MMSE. However, education had virtually no shared variance with WTAR/NART and MMSE that was not accounted for by age 11 IQ. In other words, education did not make a contribution to the association between WTAR/NART and MMSE beyond that made by age 11 IQ alone. These results support the hypothesis that the relationship between the reading tests and MMSE is driven by the sensitivity of both these types of tests to prior cognitive ability (Crawford *et al.* 2001). This was demonstrated here for the first time for the WTAR.

Of course the fact that age 11 IQ is correlated with MMSE at age 70 might mean one of two things: either MMSE scores, which measure cognitive ability in old age, are related to cognitive ability at age 11 (i.e. less intelligent children go on to have lower MMSE scores in old age that do not indicate pathology); or age 11 IQ predicts the likelihood of suffering cognitive impairment in old age (i.e. less intelligent children are more likely to suffer impairment, which is reflected in a low MMSE score). It remains to be tested how the differences in MMSE that are related to childhood IQ translate into clinical diagnoses.

## Implications for research and clinical practice

The remarkable finding from this study might be how much of MMSE–WTAR/NART association can be attributed to individual differences in a 45-min paper-and-pencil test taken at age 11. This requires further investigation, as it may have implications for both research and clinical practice. It suggests that it must be recognized how much MMSE indicates prior ability in addition to cognitive decline. This was previously shown in ‘developmentally delayed’ adults (Myers, 1987). Here, we demonstrated that scores from MMSE administered in old age are correlated with childhood cognitive ability estimates, even in a cognitively normal sample (in fact, the LBC1936 sample used in this study is cognitively higher, on average, than the population). Practitioners using MMSE should be aware of the extent to which its scores represent prior ability. What follows is that any given MMSE score does not indicate the same amount of cognitive pathology for people who have different prior cognitive ability. Depending on whether the aim is to determine the level of cognitive functioning, in absolute terms, or to infer a possible negative change from one's pre-existing level, an adjustment to the MMSE score might be advisable. The latter is often preferred in clinical contexts because it is the within-person decline that indicates a potential pathology and it is the subjective change, specific to each individual that will negatively affect their well-being, level of functioning, etc. To that end, our results suggest that MMSE cut-offs should be adapted, based on the individual's initial ability. Because they are rarely available, WTAR or NART scores might be an acceptable, relatively easily obtainable proxy for the actual pre-existing ability level. Our data indicate that an MMSE score of 22 or 23, below the commonly used cut-off (<24) might be expected from cognitively healthy older people with below average IQ. On the other hand, a score as high as 26 among people with high IQ may already be indicative of cognitive deterioration. Our cut-off for high NART scorers is in agreement with a cut-off for dementia screening among highly educated individuals, suggested elsewhere (O'Bryant *et al.* 2008)

A second finding with a potential clinical relevance and requiring further investigation is the small remaining correlation between the premorbid ability tests and the MMSE after adjustment for prior ability. This might mean that the reading tests are somewhat sensitive to cognitive impairment. However, the more likely explanation is that the degree of attenuation of the relationship between MMSE and the reading tests by controlling for prior ability is an underestimate. For example, whatever the MMSE and WTAR/NART share with respect to prior ability may not be fully captured by the MHT, because the crystallized ability captured by the NART/WTAR peaks in adulthood, a considerable time after the MHT was administered. We expect that if we had a measure of prior ability that was taken in early or middle adulthood and based on a more extensive set of tests, the attenuation might be higher or even near-to-complete. Studies with a premorbid/prior ability estimate from middle age, close to reaching their maximum crystallized ability but before any age-related cognitive decline is expected, might make a valuable contribution.

## Strengths and limitations

The principal strength of the study is the availability of a large sample of older adults, from a single year-of-birth cohort, for whom cognitive ability was tested in childhood as well as in older age. The association of WTAR/NART, education and MMSE might vary as a function of age, both because more neurodegenerative problems become apparent with ageing, and because of cohort differences in education. The narrow-age cohort used in our study provides a natural control for these potential effects. The availability of validated IQ-type test results from age 11 enabled us to test which associations found in later life stem from individual differences in cognitive ability in childhood. This was also an ideal time for the IQ test, because it is at the end of universal primary Scottish education, prior to differences in subjects being taken by different individuals in secondary education. Finally, a 6-year follow-up enabled a sensitivity analysis, excluding participants who, after the initial testing occasion, developed cognitive impairment indicative of a possible pathology.

The sample used in this study is of above-average intelligence. Our results might not generalize to people with very little education or lower cognitive ability. Given this limitation, we did not extrapolate predicted MMSE scores beyond the lowest WTAR and NART scores observed in the sample. Another limitation is the lack of prior ability measures from early to mid-adulthood. The cohort was not tested between ages 11 and 70 and so, we could only approximate their maximal prior ability from a short cognitive ability test at age 11 and from the reading tests administered at age 70. It would be useful to investigate the associations addressed in this study on a sample for whom cognitive ability data from mid-life is available.

## Conclusions

In this study, we demonstrated that a large proportion of WTAR's and NART's correlations with MMSE is explained by childhood IQ and that education does not make an additional contribution to that association, beyond that of childhood cognitive ability. This was demonstrated for the first time for the WTAR. Scores on the MMSE administered at age 70 were moderately positively correlated with cognitive abilities in childhood, suggesting that the same MMSE score might indicate different amount of cognitive pathology depending on prior cognitive ability. Where MMSE is intended as a screening tool for possible dementia or other cognitive disorder, it might be advisable to consider the obtained score in the context of prior ability, or WTAR/NART score, which can be used as its proxy.

## References

[ref1] Crawford JR, Deary IJ, Starr J, Whalley LJ (2001). The NART as an index of prior intellectual functioning: a retrospective validity study covering a 66-year interval. Psychological Medicine 31, 451–458. 11305853 10.1017/s0033291701003634

[ref2] Crawford JR, Moore JW, Cameron IM (1992). Verbal fluency: a NART-based equation for the estimation of premorbid performance. British Journal of Clinical Psychology 31, 327–329. 1393161 10.1111/j.2044-8260.1992.tb00999.x

[ref3] Deary IJ, Gow AJ, Pattie A, Starr JM (2012). Cohort profile: the lothian birth cohorts of 1921 and 1936. International Journal of Epidemiology 41, 1576–1584. 22253310 10.1093/ije/dyr197

[ref4] Deary IJ, Gow AJ, Taylor MD, Corley J, Brett C, Wilson V, Campbell H, Whalley LJ, Visscher PM, Porteous DJ, Starr JM (2007). The lothian birth cohort 1936: a study to examine influences on cognitive ageing from age 11 to age 70 and beyond. BMC Geriatrics 7, 28. 18053258 10.1186/1471-2318-7-28 PMC2222601

[ref5] Deary IJ, Whalley LJ, Crawford JR (2004). An ‘instantaneous’ measure of a lifetime's cognitive change. Intelligence 32, 113–119.

[ref6] Dykiert D, Deary IJ (2013). Retrospective validation of WTAR and NART scores as estimators of prior cognitive ability using the Lothian Birth Cohort 1936. Psychological Assessment 25, 1361–1366. 23815111 10.1037/a0033623

[ref7] Folstein MF, Folstein SE, McHugh PR (1975). Mini-mental state: a practical method for grading the cognitive state of patients for the clinician. Journal of Psychiatric Research 12, 189–198. 1202204 10.1016/0022-3956(75)90026-6

[ref8] Green REA, Melo B, Christensen B, Ngo L-A, Monette G, Bradbury C (2008). Measuring premorbid IQ in traumatic brain injury: an examination of the validity of the Wechsler Test of Adult Reading (WTAR). Journal of Clinical and Experimental Neuropsychology 30, 163–172. 18213530 10.1080/13803390701300524

[ref9] Horn JL, Cattell RB (1967). Age differences in fluid and crystallized intelligence. Acta Psychologica 26, 107–129. 6037305 10.1016/0001-6918(67)90011-x

[ref10] McFarlane J, Welch J, Rodgers J (2006). Severity of Alzheimer's disease and effect on premorbid measures of intelligence. British Journal of Clinical Psychology 45, 453–463. 17076957 10.1348/014466505X71245

[ref11] McGurn B, Starr JM, Topfer JA, Pattie A, Whiteman MC, Lemmon HA, Whalley LJ, Deary IJ (2004). Pronunciation of irregular words is preserved in dementia, validating premorbid IQ estimation. Neurology 62, 1184–1186. 15079021 10.1212/01.wnl.0000103169.80910.8b

[ref12] Myers B (1987). The mini mental state in those with developmental disabilities. Journal of Nervous and Mental Disease 175, 85–89. 2433389 10.1097/00005053-198702000-00003

[ref13] Nelson HE, McKenna P (1975). The use of current reading ability in the assessment of dementia. British Journal of Social and Clinical Psychology 14, 259–267. 1182406 10.1111/j.2044-8260.1975.tb00178.x

[ref14] Nelson HE, Willison J (1991). National Adult Reading Test *(*NART*)* Manual, 2nd edn. NFER-Nelson: Windsor, UK.

[ref15] O'Bryant SE, Humphreys JD, Smith GE, Ivnik RJ, Graff-Radford NR, Petersen RC, Lucas JA (2008). Detecting dementia with the mini-mental state examination (MMSE) in highly educated individuals. Archives of Neurology 65, 963–967. 18625866 10.1001/archneur.65.7.963 PMC2587038

[ref16] O'Carroll RE, Prentice N, Murray C, Van Beck M, Ebmeier KP, Goodwin GM (1995). Further evidence that reading ability is not preserved in Alzheimer's disease. British Journal of Psychiatry 167, 659–662. 8564324 10.1192/bjp.167.5.659

[ref17] Patterson K, Graham N, Hodges JR (1994). Reading in dementia of the Alzheimer type: a preserved ability? Neuropsychology 8, 395–407.

[ref18] Psychological Corporation (2001). Wechsler Test of Adult Reading. The Psychological Corporation: San Antonio, TX.

[ref19] R Core Team (2015). R: A Language and Environment for Statistical Computing. R Foundation for Statistical Computing: Vienna, Austria http://www.R-project.org/

[ref20] Revele W (2015). Procedures for Personality and Psychological Research. Northwestern University: Evanston, Illinois, USA.

[ref21] Riley GA, Simmonds LV (2003). How robust is performance on the National Adult Reading Test following traumatic brain injury? British Journal of Clinical 42, 319–328. 10.1348/01446650360703410 14565896

[ref22] SCRE (1933). The Intelligence of Scottish Children: A National Survey of an Age-Group. Scottish Council for Research in Education. University of London Press: London, UK.

[ref23] SCRE (1949). The Trend of Scottish Intelligence: A Comparison of the 1947 and 1932 Surveys of the Intelligence of Eleven-Year-Old Pupils. Scottish Council for Research in Education. University of London Press: London, UK.

[ref24] Starr JM, Whalley LJ, Inch S, Shering PA (1992). The quantification of the relative effects of age and NART-predicted IQ on cognitive function in healthy old people. International Journal of Geriatric Psychiatry 7, 153–157.

[ref25] Stebbins GT, Wilson RS, Gilley DW, Bernard BA, Fox JH (1990). Use of the national adult reading test to estimate premorbid IQ in dementia. Clinical Neuropsychologist 4, 18–24. 10.1080/13854049008401493 29022436

[ref26] Steiger JH (1980). Tests for comparing elements of a correlation matrix. Psychological Bulletin 87, 245–251.

[ref27] Tombaugh TN, McIntyre NJ (1992). The Mini-Mental State Examination: a comprehensive review. Journal of the American Geriatrics Society 40, 922–935. 1512391 10.1111/j.1532-5415.1992.tb01992.x

